# Impact of biometeorological conditions and air pollution on influenza-like illnesses incidence in Warsaw

**DOI:** 10.1007/s00484-021-02076-2

**Published:** 2021-01-17

**Authors:** Katarzyna Lindner-Cendrowska, Peter Bröde

**Affiliations:** 1 Institute of Geography and Spatial Organization, Polish Academy of Sciences, Twarda 51/55, 00-818 Warsaw, Poland; 2 Leibniz Research Centre for Working Environment and Human Factors at TU Dortmund (IfADo), Dortmund, Germany

**Keywords:** Influenza, Influenza-like illness, Air quality, *UTCI*, Seasonality, Biometeorology

## Abstract

In order to assess the influence of atmospheric conditions and particulate matter (PM) on the seasonally varying incidence of influenza-like illnesses (ILI) in the capital of Poland—Warsaw, we analysed time series of ILI reported for the about 1.75 million residents in total and for different age groups in 288 approximately weekly periods, covering 6 years 2013–2018. Using Poisson regression, we predicted ILI by the Universal Thermal Climate Index (*UTCI*) as biometeorological indicator, and by PM2.5 and PM10, respectively, as air quality measures accounting for lagged effects spanning up to 3 weeks. Excess ILI incidence after adjusting for seasonal and annual trends was calculated by fitting generalized additive models. ILI morbidity increased with rising PM concentrations, for both PM2.5 and PM10, and with cooler atmospheric conditions as indicated by decreasing *UTCI*. While the PM effect focused on the actual reporting period, the atmospheric influence exhibited a more evenly distributed lagged effect pattern over the considered 3-week period. Though ILI incidence adjusted for population size significantly declined with age, age did not significantly modify the effect sizes of both PM and *UTCI*. These findings contribute to better understanding environmental conditionings of influenza seasonality in a temperate climate. This will be beneficial to forecasting future dynamics of ILI and to planning clinical and public health resources under climate change scenarios.

**Supplementary Information:**

The online version contains supplementary material available at 10.1007/s00484-021-02076-2.

## Introduction

Influenza is a seasonal, highly contagious virus that causes periodical epidemics around the world. Each year, an estimated 1 billion cases of influenza is reported, many of which result in severe complications such as pneumonia or even in respiratory deaths (4.0–8.8 fatalities per 100,000 individuals annually) (Iuliano et al. 2018). The virus is known to spread differentially across age groups (Mossong et al. 2008; Huang et al. 2017). Very young children (0–4 years old) and the elderly are reckoned as high-risk groups that suffer from influenza-related complications most often (Ruf and Knuf 2014; Li et al. 2018). Influenza is transmitted between individuals via direct contact with secretions (by contact with contaminated host or surface) or large droplets (through coughing and sneezing), as well as through airborne transmission (by inhaling small aerosol droplets containing virions that remain suspended in the air) (Shaman and Kohn 2009; Tamerius et al. 2011). The incubation period of influenza is short and ranges from 1 to 4 days (Cox and Subbarao 1999).

Human influenza viruses are divided into three genera—influenza A, B, and C, from which only A and B types cause epidemics in humans (WHO 2020). Thanks to vaccination or as a result of prior infection, population gains immunity to currently spreading strain of virus. However, the influenza virus, espiecially type A, tends to evolve commonlly, using mechanisms like antigenic “drift” or less frequent, but more substantial antigenic “shift” (National Research Council 2001; Nelson and Holmes 2007; Bouvier and Palese 2008). Therefore, vaccinated or recently recovered individuals are usually suscepible to new influenza strains that can emerge in the population (Yaari et al. 2013). Although there are many theoretical and experimental studies on the evolution of influenza virus, the timing and properties of new antigenic clusters remain unpredictable (Petrova and Russell 2018).

In temperate climates, influenza morbidity has a clear seasonal pattern. The virus epidemics occur from late autumn to the beginning of spring, with largest peaks in winter (Cox and Subbarao 2000; Heikkinen and Järvinen 2003; Finkelman et al. 2007; Moorthy et al. 2012). These outbreaks are synchronized between regions and countries that have similar climatic conditions (Crépey and Barthélemy 2007; Wenger and Naumova 2010; Schanzer et al. 2011) and usually last from 1 to 4 months (Simonsen 1999). For the rest of the year, influenza stays active at very low level (Lofgren et al. 2007). On the contrary in tropical and subtropical climatic zones, temporal patterns of virus activity are more complex and frequently associated with rain season (de Arruda et al. 1991; Moura et al. 2009). Many hot and humid regions experience two distinct epidemical seasons within the year: in winter and summer/autumn (Chan et al. 2009; Tamerius et al. 2011; Wang et al. 2017). Although influenza incidence exhibits strong seasonality, the magnitude and timing of its outbreaks change from year to year even within one population (Huppert et al. 2012; Moorthy et al. 2012; Yaari et al. 2013).

There is no single recognized main driver of influenza seasonality (Shaw Stewart 2016); however, three mechanisms may explain viral seasonality in temperate climate (Lipsitch and Viboud 2009; Tamerius et al. 2011; Pica and Bouvier 2012), as described in detail below:

1. *Seasonal variations in host contact rate*: With decreasing ambient temperature in winter, people spend more time crowding indoors, which favours greater disease transmission (Lofgren et al. 2007; Fisman 2012). However, studies in mice indicated that even under identical crowding conditions, flu transmission demonstrates a clear seasonal pattern (Schulman and Kilbourne 1963). Thus, temperature-dependent changes in host contact rates are not the main factor, but rather contribute to the seasonal incidence of influenza by amplifying transmission among population (Lofgren et al. 2007; Shaw Stewart 2016).
2. *Virus survival capability and effectiveness of transmission*: Thermal conditions have also important impact on the survival of influenza virus. In colder environment, the integrity of viral envelope is better, whereas at higher air temperature, above 21 °C, the envelope becomes disordered, leading to higher virus susceptibility to damage (Polozov et al. 2008). With seasonal rise of air temperature, virions can stay dormant in lower parts of human respiratory tract until late autumn and winter, when sudden chilling of inhaled air frequently occurs, which activates dormant viruses and starts an infection as a result (Shaw Stewart 2016). Seasonally changing influenza virulence is also dependent on its possibility to survive outside human body and to transmit effectively from one host to another. The most sensitive to atmospheric conditions is airborne transmission (Lowen et al. 2007). At low relative humidity (RH—20–35%), large droplets, expelled during coughing or sneezing, evaporate quickly, leading to creation of smaller and lighter droplets that are more likely to remain suspended in the air for an extended period of time (Schaffer et al. 1976; Tellier 2006; Weber and Stilianakis 2008). Therefore airborne transmission of influenza is the most efficient in cold dry air (Weinstein et al. 2003; Lowen and Steel 2014; Gustin et al. 2015).
3. *Fitness of the host immune system*: There is a substantial evidence that seasonal variations of influenza incidence may be the effect of cyclically changing host immunity to respiratory tract illnesses (Dowell 2001). Human susceptibility to viral infections changes with photoperiod variations (Lofgren et al. 2007). In wintertime, due to deficiency of UV radiation, a lower vitamin D production in the organism is observed, which results in immune suppression and lower ability to fight infections (Cannell et al. 2006; Ginde et al. 2009; Urashima et al. 2010; Martineau et al. 2017). Increased host susceptibility to viruses is also linked to seasonal decrease of air temperature. Inhaling cold air and excess cooling of the body surface can induce cold stress that leads to pathophysiological responses of human organism that compromise immune system (Mourtzoukou and Falagas 2007). Breathing dry and cold air may cause constriction of the blood vessels of the upper airways, as well as desiccation of nasal mucosa and reduction of mucociliary clearance, all of which result in increased susceptibility to infection (Salah et al. 1988; Eccles 2002). Moreover, excessive chilling increases energy demand for thermoregulation that limits energy available for immune defence and leaves human organism vulnerable to pathogens (Lochmiller and Deerenberg 2000; Lofgren et al. 2007; Jaakkola et al. 2014).

Although local meteorological factors clearly affect transmission of virus infections (Fdez-Arroyabe 2012; Price et al. 2019), our understanding of the role of specific climatic determinants in influenza epidemiology is very limited and many uncertainties still remain (Pica and Bouvier 2012). There is a general agreement that high sun activity (especially intensive UV radiation) inactivates enveloped viruses (Jensen 1964; Sagripanti and Lytle 2007). Mean air temperature is also an important factor affecting influenza epidemics in temperate climate (du Prel et al. 2009; Sundell et al. 2016). Viral infectivity and persistence are higher in colder environments (Lofgren et al. 2007; Lowen et al. 2007), while laboratory tests confirm that at temperature above 30 °C, influenza transmission in aerosol stops completely (Lowen et al. 2008). However, a study from Northern Finland demonstrates that the risk of infection paradoxically can decrease also at very low air temperature (Jaakkola et al. 2014). Furthermore, Shaw Steward (2016) and Li et al. (2018) argued that it is not absolute temperature value, but rather diurnal temperature variations that are responsible for higher acute respiratory tract infections incidence. There are inconclusive results about the potential impact of air humidity on influenza morbidity. Numerous studies confirmed that low content of water vapour in the air increases the transmission and survival of aerosolized influenza virions (Tellier 2006; Brankston et al. 2007; Lowen et al. 2007). Conversely, other studies indicate that higher influenza activity is rather associated with high relative humidity (Chan et al. 2009; Li et al. 2018). On the other hand, Tang et al. (2010) and Iha et al. (2016) emphasize that depending on the influenza type (A or B) and geographical location, the correlation between infection rate and RH may be positive or negative. However, some researchers (Shaman and Kohn 2009; McDevitt et al. 2010; Pica and Bouvier 2012) suggest that high survival and transmission rates of influenza show stronger association with absolute humidity (AH). Low AH values highly correlate with the onset of influenza epidemics in USA (Shaman et al. 2010) and Japan (Shoji et al. 2011). Less attention has been paid to the effects of other meteorological factors on influenza incidence so far. Several previous studies showed low wind speed contributing to virus spread (Schulman and Kilbourne 1963; Xiao et al. 2013; Roussel et al. 2016). Precipitation has been reckoned to be insignificant as influenza predictor (Tang et al. 2010) or otherwise was associated with respiratory tract infections morbidity, especially in the tropics (Chan et al. 2002; Robertson et al. 2004; Agrawal et al. 2009; Gomez-Barroso et al. 2017).

There is also some evidence that poor air quality can be a significant risk factor for lower respiratory tract infections. Exposure to atmospheric pollution leads to irritation and mechanical damage of airway mucosa, due to generation of free radicals, compromising mucociliary clearance and diminishing individual resistance to viral infections (Ciencewicki and Jaspers 2007; Fuhrmann 2010). Moreover, laboratory tests indicated that transport pollutants (precisely diesel exhaust) increase the ability of influenza virus to attach and enter to respiratory epithelial cells (Jaspers et al. 2005). Specifically, particulate matter is frequently associated with higher morbidity due to respiratory infections (Hwang and Chan 2002; Chen et al. 2017). PM10 contributes to the transmission of influenza by providing condensation nuclei to which virus droplets can attach and therefore remain suspended in the air for time long enough to be inhaled by a susceptible individual (Hammond et al. 1989; Lee et al. 2014; Feng et al. 2016).

Determining the presence of the influenza virus in the population, by performing laboratory tests, is costly, thus performed occasionally by random sampling on symptomatic patients, in order to determine current circulating strains (Meerhoff et al. 2004). Furthermore, influenza can be difficult to diagnose basing on clinical symptoms alone, as various other viruses and bacteria can cause similar health ailments (Heikkinen and Järvinen 2003; Eccles 2005; Liu et al. 2019), and only 5–15% of common colds is induced by influenza virus (Zambon et al. 2001). Alternatively, a common respiratory syndrome, called influenza-like illness (ILI), frequently serves as influenza-proxy in epidemiological studies (Mandl et al. 2004; Minh An et al. 2014; Silva et al. 2014; Su et al. 2019). Although the commonly weekly reported number of ILI cannot exactly correspond to real influenza morbidity, it can generally represent the influenza activity in the population (Wang et al. 2017). Recent studies have shown that laboratory confirmed influenza cases were highly correlated with ILI rates (Yaari et al. 2013). In addition, applying a weekly time-scale enables overcoming the problem of under representativeness of ILI cases during weekends and public holidays, due to limited accessibility to healthcare institutions (Buckingham-Jeffery et al. 2017). This approach also compensates for the increased daily admissions on the first labour day after the break, described earlier as “the Monday effect” (Romaszko et al. 2019).

Although many previous studies have recognized the significance of meteorological parameters on the transmission and influenza morbidity, the results differ regionally and are strongly affected by the climate type and social structure. In Poland, the association between weather variables and viral infections was so far analysed in detail only in relation to general respiratory tract diseases (Romaszko et al. 2019).

Therefore, the aim of the current study is to determine whether seasonal variability of influenza-like illnesses is affected by atmospheric conditions and air quality in the capital of Poland—Warsaw.

## Materials and methods

Warsaw, with a population of 1.78 million (Statistics Poland 2020), is situated in the central part of the country, on the Masovian Plain. It is characterized by humid continental climate (Dfb type), with cold, cloudy winter, and warm summer (Peel et al. 2007), which is modified by the Urban Heat Island effect (Błażejczyk et al. 2014). Mean monthly temperature fluctuates in Warsaw from − 1.9 °C in January to 19.0 °C in July, while yearly rainfall total is 531.5 mm (Institute of Meteorology and Water Management 2020).

### Epidemiological data

Data on influenza incidence was provided by the Voivodship Unit of the State Sanitary Inspection in Warsaw. Polish medical centres report to the State Sanitary Inspection weekly rates of ILI incidence, based on a clinical case definition. ILI cases are defined as sudden onset of at least one general symptom (fever or subfebrile condition, malaise, headache, muscle pain) followed by at least one of the respiratory symptoms (cough, sore throat, dyspnoea) (Department of Infectious Disease Epidemiology and Surveillance NIZP-PZH 2019). This definition is in general consistent with J10 and J11 codes in ICD-10 classification.

It is important to notice that reporting periods in Polish surveillance system are not always 7-day periods, i.e. 1 week exactly. In each month, first and third period consist of 7 days, the second one is 8 days long, while the forth reporting period varies from 6 to 9 days. Thus, the time series consisted of 4 periods per month, 48 periods per year, and 288 periods covering 6 years from 1 January 2013 to 31 December 2018. To overcome the unbalanced number of days per period, mean daily ILI incidence (dILI) was calculated, dividing total amount of ILI cases by the number of days for each reporting period, while adjusting to population size (cf. below). Influenza incidence reports in Poland do not comprise any personal information, like gender or accompanying chronic diseases, but total ILI cases are subdivided into four age groups: 0–4 (infants and young children), 5–14 (school children), 15–64 (youngsters and adults), and above 64 years old (elderly).

To account for changing population size, yearly data on the number of Warsaw residents corresponding to the observation period (2013–2018) and to the four age groups were obtained from Local Data Bank of Polish Central Statistical Office (Statistics Poland 2020).

### Meteorological and air quality data

In order to characterize typical meteorological conditions in Warsaw (Poland), meteorological data from Warsaw-Bielany weather station (52.17 N, 20.58 E, 98 m a.s.l.) was obtained from the Institute of Meteorology and Water Management in Warsaw (IMGW), Poland. This weather station was selected due to high completeness of the data set and its representativeness for the bioclimate of the build-up areas within the city. Hourly air temperature (*t*, °C), vapour pressure (*vp*, hPa), relative humidity (*RH*, %), wind speed (*v*, m/s) and cloudiness (*N*, octas), were collected from the period of analysis, i.e. 2013–2018.

Thermal stress causes significant load on human organism and increases energy demand for thermoregulation at the expense of energy available for immune defence, which may result in higher susceptibility to infections (Lochmiller and Deerenberg 2000). To assess biothermal environment and degree of thermal stress in men, the Universal Thermal Climate Index (*UTCI*) was applied. *UTCI* is defined as an equivalent ambient temperature (°C) of a reference environment, causing the same physiological response of a reference human organism as the actual environment (Błażejczyk et al. 2013). Hourly values of *UTCI* were calculated in BIOKLIMA 2.6 software (Błażejczyk 2005). In order to obtain *UTCI* values, the software calculates mean radiant temperature (*tmrt*, °C), using following formula (Eq. 1):

1 $$ tmrt={\left(\frac{\frac{R}{Irc}+0.5\cdotp Lg+0.5\cdotp La}{s_h\cdotp \sigma}\right)}^{0.25}-273 $$

where *R* is the absorbed solar radiation (W/m^2^), *Irc* is the coefficient reducing convective and radiative heat transfer through clothing, *Lg* is the ground radiation (W/m^2^), *La* is the atmosphere outgoing radiation (W/m^2^), s_h_ is the emissivity coefficient for humans (0.95), and σ is the Stefan-Boltzmann constant (5.667/10^8^ W/m^2^·K^4^). Absorbed solar radiation (*R*) was calculated from cloudiness (*N*) using the SolAlt model provided by BIOKLIMA. Detailed formulas are available online in Electronic Supplemental Note 1.

Hourly means of PM2.5 and PM10 concentration were obtained from the one of the Chief Inspectorate of Environmental Protection (GIOŚ) air quality monitoring points—Warszawa-Ursynów (52.16 N, 21.03 E, 102 m a.s.l.). Air pollution data originated only from the one place within Warsaw agglomeration, as it was the only station that recorded detailed measurements of PM2.5 and PM10 over a long enough period of time.

All meteorological and air pollution data, as well as biometeorological indices, were averaged and aligned to the ILI reporting periods.

### Statistical analysis

Descriptive statistical analyses were conducted to describe the nature of weekly rates of medical consultations due to influenza-like illnesses, as well as meteorological and aerosanitary data. Considering the potential non-Gaussianity of both meteorological variables and air pollutants data, Spearman correlation was used to illustrate the association between ILI and particular weather parameters and the air pollutants.

**Fig. 1:**
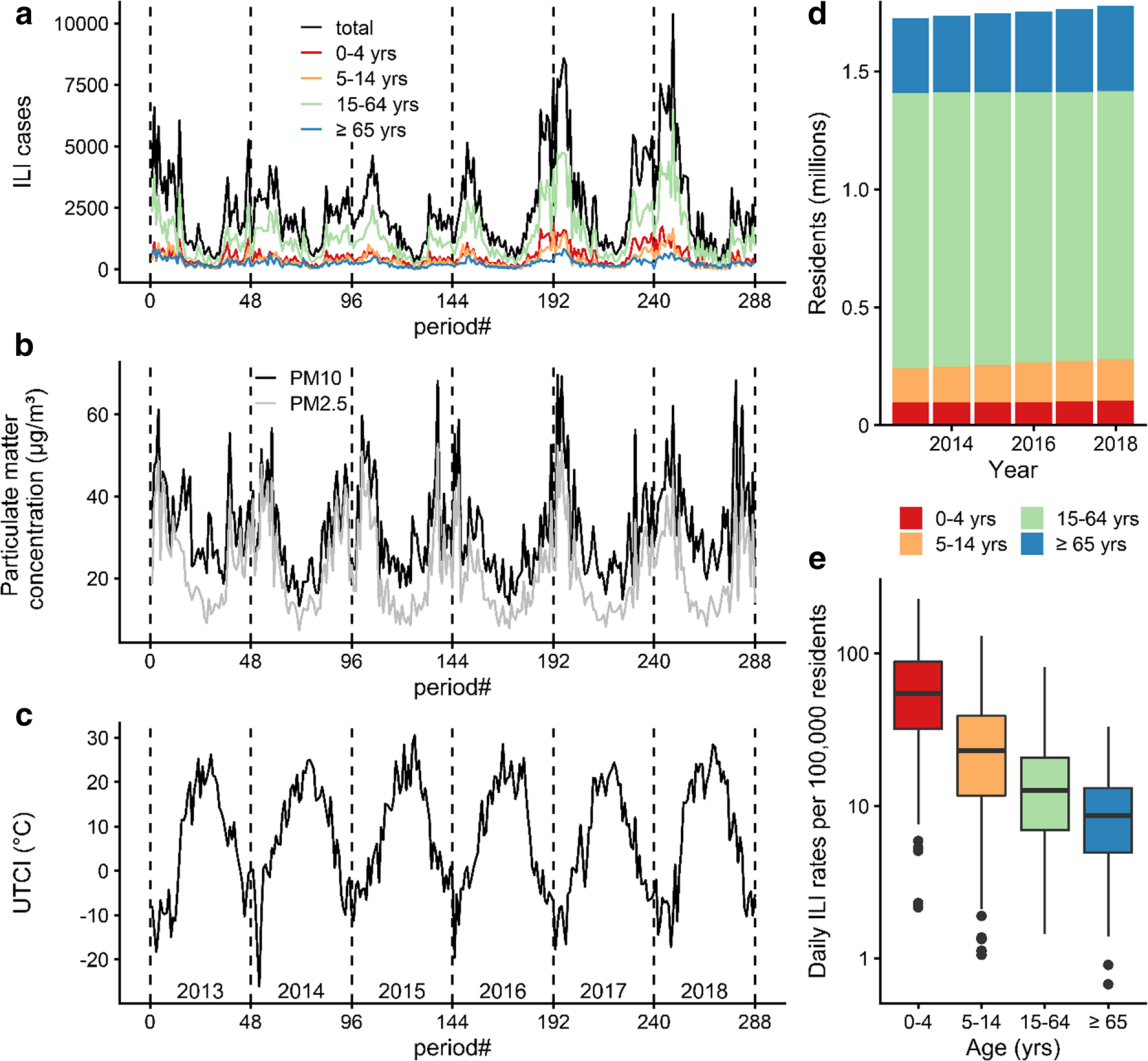
Time series of ILI cases in total and in different age strata (**a**), of PM2.5 and PM10 (**b**), and of *UTCI* (**c**) over the 288 reporting periods covering the time segment from 01 January 2013 to 31 December 2018 with dashed vertical lines indicating the end of the reporting year. Annual development of Warsaw population stratified by age (**d**), and log-scaled distribution of daily ILI rates per 100,000 residents related to age (**e**)

Following a recently described modelling framework (Bhaskaran et al. 2013), we analysed the influence of *UTCI* and PM2.5, as well as *UTCI* and PM10, respectively, on excess ILI incidence, i.e. after adjusting for seasonal and long-term trends. We applied Poisson regression models allowing for over-dispersion separately to the time series for the different age groups as well as for the total numbers (Fig. 1).

Fitting generalized additive models (GAM), the log ILI counts at period *t* (*t* = 1,…,288) were predicted in separate models according to Eq. 2 by linear terms for either PM2.5 or PM10 as indicators of aerosanitary conditions, adjusting for the biometeorological influence represented by *UTCI*. In addition, penalized cubic regression splines (Wood 2006) were included as predictors of the seasonal cycle within a year (*f*_*seas*_) and the long-term annual trend (*f*_*long*_), respectively. Specifically, using the notation introduced by Wood (2006), *f*_*long*_ refers to a natural cubic regression spline with basis dimension set to *k* = 9 (i.e. with *k*–1 = 8 as upper limit of the associated degrees of freedom). Similarly, *f*_*seas*_ represents a cyclic cubic regression spline with *k* = 12 applied to the period-within-year calculated as *t mod* 48 + 1.

2 $$ \log \left({ILI}_t\right)= intercept+\alpha\ {UTCI}_t+\beta\ {PM}_t+{f}_{seas}\left(t\ \mathit{\operatorname{mod}}\ 48+1\right)+{f}_{long}(t)+{\gamma}_{\equiv 1}\log \left({pt}_t\right) $$

In order to consider the varying population size and lengths of the reporting periods, ILI counts were analysed as rates, i.e. as daily ILI per unit population size. This was achieved by including the log product of population size multiplied with days within the reporting period (*pt*), as offset with coefficient *γ*_*≡1*_ fixed to one. This approach is a commonly applied statistical technique to treat such data within the Poisson regression framework (Dunn and Smyth 2018). Thus, the estimated coefficients *α* of *UTCI* and *β* of PM2.5 or PM10 in Eq. 2 represent log rate ratios (RR), from which we calculated the percentage change in daily ILI incidence rate per unit increase in *UTCI* or *PM*, respectively, as %change = (RR-1)·100.

In previous studies, 7 to 14 days delay between weather change and increase in respiratory tract infections incidence has been observed (Nastos and Matzarakis 2006; Mäkinen et al. 2009; Sundell et al. 2016). To investigate the lag effect between particular biometeorological and aerosanitary conditions and ILI incidence in Warsaw, we considered values of *UTCI*, PM2.5, and PM10 lagged by one and two reporting periods in addition to the actual value (lag 0). Following the approach suggested by Bhaskaran et al. (2013), we fitted individual lag models, i.e. separate models including as predictors the different lagged (0, 1, and 2 periods) values of *UTCI*, PM2.5, and PM10, as well as the averaged lagged values, respectively.

We compared these models to a distributed lag model (Eq. 3) considering all lagged values simultaneously, from which we calculated the cumulative or “net” effect over all lags as the sum of the coefficients of the different lagged exposures (Bhaskaran et al. 2013).

3 $$ \log \left({ILI}_t\right)= intercept+{\sum}_{l=0}^2{\alpha}_l{UTCI}_{t-l}+{\sum}_{l=0}^2{\beta}_l{PM}_{t-l}+{f}_{seas}\left(t\ \mathit{\operatorname{mod}}\ 48+1\right)+{f}_{long}(t)+{\gamma}_{\equiv 1}\log \left({pt}_t\right) $$

In order to investigate the interaction of age with the effects of PM2.5, PM10 and *UTCI* on excess ILI incidence after adjusting for seasonal and long-term trends, respectively, we additionally performed the above described analysis with the averaged lagged values as predictors after combining the series of the four groups and introducing age as additional factor in Eq. 2 (Wood 2006).

The calculations were carried out with version 3.6.3 of the R software (R Core Team 2020) using packages mgcv (Wood 2006), mgcViz (Fasiolo et al. 2020), and emmeans (Lenth 2020).

## Results

A total of 713,399 ILI cases were reported in Warsaw from January 2013 to December 2018. The descriptive statistics for meteorological and air pollution weekly data and mean daily ILI morbidity rates (total and in different age groups) per 100,000 residents were summarized in Table 1. The mean ILI morbidity per 100,000 residents was 18.8 per day, with the maximum of 83.3 from 1 to 7 March 2018. During the study period, meteorological conditions were typical for the Dfb (cold with warm summer) Köppen climate zone (Peel et al. 2007). Mean weekly *UTCI* values varied from − 26.0 °C in January 2014 to 30.5 °C in August 2015, what corresponds to *UTCI* categories from strong cold stress to moderate heat stress. The PM2.5 and PM10 daily concentrations varied seasonally and reached maximum values usually in the cold season (Fig.1b), as air pollution in Poland is mainly related to residential heating systems (Adamczyk et al. 2017) using individual, coal-fired heating stoves.

**Table 1:** Descriptive statistics for weekly atmospheric data and influenza-like illness (total and in age groups) from 288 reporting periods in Warsaw (2013–2018)

Variables	Mean ± SD	Min	Percentile			Max
			25	50	75	
Atmospheric data						
Air temperature *t* (°C)	10.7 ± 8.6	− 10.7	3.5	10.7	18.4	28.2
Relative humidity *RH* (%)	72.3 ± 12.6	36.8	62.6	72.3	83.1	93.9
Vapour pressure *vp* (hPa)	9.9 ± 4.2	1.9	6.4	9.4	13.4	21.1
Wind speed *v* (m/s)	2.4 ± 0.6	1.2	1.9	2.3	2.8	4.2
Mean radiant temperature *tmrt* (°C)	11.5 ± 14.4	− 18.7	− 1.4	11.4	24.4	39.8
*UTCI* (°C)	7.4 ± 12.3	− 26.0	− 3.1	7.7	18.4	30.5
PM2.5 (μg/m^3^)	21.8 ± 10.4	7.4	13.4	18.9	28.6	52.8
PM10 (μg/m^3^)	31.6 ± 11.5	13.2	23.1	29.0	38.5	69.7
Daily influenza-like illness (*dILI*) per 100,000 residents						
0–4 years old	67.9 ± 49.7	2.2	31.9	54.9	88.5	228.5
5–14 years old	29.7 ± 24.6	1.1	11.7	22.9	39.0	130.4
15–64 years old	15.8 ± 12.3	1.4	7.0	12.6	20.8	81.9
65 years old and above	9.9 ± 6.3	0.7	4.9	8.6	13.1	33.3
Total	18.8 ± 13.8	1.6	9.0	15.3	24.9	83.3

Note: *SD* standard deviation

The time series of ILI counts (Fig. 1a) exhibited a seasonal pattern with maximum values during winter and minimum numbers during summer, as well as demonstrated annual differences with the highest daily ILI rates in 2018 and 2017, respectively. This periodicity was observed for the total sample as well as for the different age groups. While the seasonal ILI pattern was similar to the course of PM2.5 and PM10 (Fig. 1b), it was inversely related to *UTCI* (Fig. 1c). The highest ILI counts were observed for people 15–64 years of age (Fig. 1a), who form the majority of Warsaw residents (Fig. 1d). When standardized for population size (Fig. 1e), daily ILI incidence rate showed a decreasing trend with age.

Total daily influenza-like illnesses (*dILI*) rates were significantly correlated with all analysed atmospheric variables (Tab. 2). The strongest negative correlation was between *dILI* and vapour pressure (*vp)* or air temperature (*t*) (both − 0.73), but also *UTCI* (− 0.72). Moderate strong positive associations were observed between *dILI* and particulate matter (0.67 for PM2.5 and 0.54 for PM10). Furthermore, air pollution concentration (especially PM2.5) was strongly negatively correlated with *t*, mean radiant temperature (*tmrt*), *UTCI*, and *vp*. Correlations between *dILI* and meteorological elements (with the exception of *v*) were the strongest for children 5–14 years, while the strongest relationship between dILI and particulate matter was observed for elderly people (≥ 65 years).

**Table 2:** Spearman correlation coefficients between *dILI* per 100,000 residents and atmospheric parameters from 288 reporting periods in Warsaw (2013–2018)

	*dILI*					*t*	*RH*	*vp*	*v*	*tmrt*	*UTCI*	*PM2.5*
	*Total*	*0–4 years*	*5–14 years*	*15–64 years*	*≥ 65 years*							
*t*	− 0.73**	− 0.68**	− 0.74**	− 0.71**	− 0.68**							
*RH*	0.44**	0.44**	0.44**	0.43**	0.43**	− 0.69**						
*vp*	− 0.73**	− 0.67**	− 0.74**	− 0.72**	− 0.67**	0.95**	− 0.45**					
*v*	0.35**	0.32**	0.29**	0.36**	0.35**	− 0.39**	0.24**	− 0.37**				
*tmrt*	− 0.69**	− 0.65**	− 0.70**	− 0.67**	− 0.64**	0.98**	− 0.76**	0.90**	− 0.37**			
*UTCI*	− 0.72**	− 0.68**	− 0.73**	− 0.71**	− 0.67**	0.99**	− 0.69**	0.93**	− 0.49**	0.98**		
*PM2.5*	0.67**	0.60**	0.66**	0.66**	0.67**	− 0.77**	0.57**	− 0.73**	0.11**	− 0.76**	− 0.74**	
*PM10*	0.54**	0.47**	0.51**	0.54**	0.56**	− 0.54**	0.32**	− 0.56**	0.04**	− 0.50**	− 0.50**	0.88**

Note: ^**^ *p* < 0.0001, ^*^ *p* < 0.05, all abbreviations are explained in Table 1

**Fig. 2:**
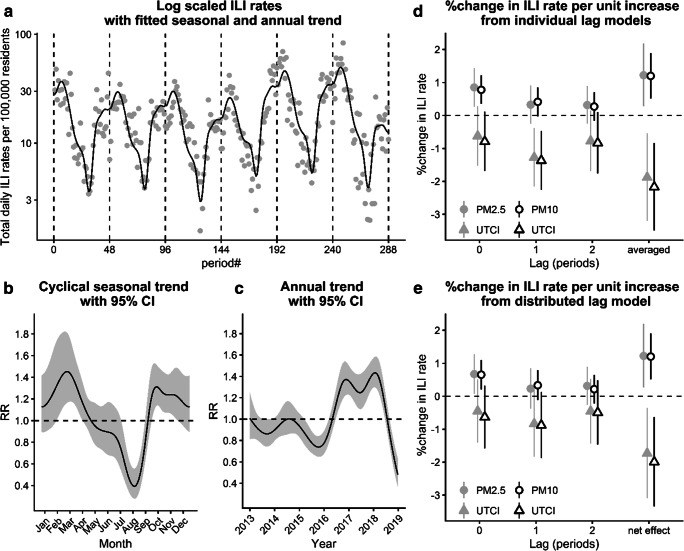
Total daily ILI rates on a log scale with dashed vertical lines indicating the end of the reporting year (**a**), with spline functions fitted by generalized additive models (GAM) to seasonal (**b**) and annual trends (**c**) expressed as rate ratios (RR) relative to the overall mean. Percentage change in ILI rates per unit increase in the predictors from Poisson regression models individually considering different lagged and the averaged lagged values of *UTCI* together with PM2.5 (grey symbols) or PM10 (black open symbols), respectively, (**d**), as well as distributed lagged and cumulated net effects (**e**). Error bands and bars indicate 95% confidence intervals (CI)

Figure 2 summarizes the results from the Poisson regression analyses performed for the time series of total daily ILI incidence rates with the trend function fitted by GAM (Fig. 2a). A cyclical seasonal component, showing a minimum in summer and a maximum for late winter (Fig. 2b), and annual fluctuations with increased rates in 2017 and 2018 and lower rates during the final year (Fig. 2c) were detected. After adjusting for general trend, the individual lag models indicated rising ILI rates per unit increase in PM2.5 or PM10 and per unit decrease in *UTCI*, respectively (Fig. 2d). Both PM2.5 and PM10 effects were statistically significant for the current period (lag 0) value and declined at higher lags. In turn, for *UTCI*, the estimates were less differentiated over the three lag periods, with the greatest, statistically significant effect at one-period-lagged values (Tab. S1 in the Electronic Supplemental Material ESM). The overall effect on ILI incidence, represented by the averaged single lag values of PM2.5 and *UTCI*, demonstrated that estimated ILI rate changes were 1.2% per 1 μg/m^3^ increase in PM2.5 and 1.9% per 1 °C decrease in *UTCI* (Fig. 2d). For the overall effect of PM10 and *UTCI*, the estimated ILI rate changes were 1.2% per 1 μg/m^3^ increase and 2.2% per 1 °C decrease, respectively. In order to demonstrate the cumulative effect of an exposure to air pollution and particular biothermal conditions over all three periods (for 0, 1, and 2 lag together), the unconstrained distributed lag model simultaneously considered all lagged variables as predictors (Fig. 2e). This model corroborated the results for individual models, although the estimates for the lagged effects were slightly reduced and statistically non-significant for *UTCI*. The smaller magnitudes of the effects and wider confidence intervals in the distributed model were presumably a result of the multi-collinearity between the lagged predictor values. The cumulative net effects, calculated from the summed coefficients of the lagged exposures (Fig. 2e), were almost identical to the effects from the individual model using the averaged lagged values as predictors (Fig. 2d). Accordingly, the estimated ILI rates also increased 1.2% per 1 μg/m^3^ increase in PM2.5 and PM10 concentrations, but were a bit smaller for 1 °C decrease in *UTCI* values (− 1.7 and 2.0%, respectively) (Tab. S2 in ESM).

We performed a sensitivity analysis to verify the linear Poisson regression approach by replacing the linear terms for *UTCI* and *PM* in Eq. 2 with penalized tensor-product splines with basis dimension set to *k* = 5 (Wood 2006) allowing for potential nonlinear relationships of excess ILI incidence with air pollution and meteorological influences after adjusting for seasonal and long-term annual trends. However, the corresponding results, e.g. as shown in Fig. S1 in ESM for the averaged lagged effects of *UTCI* and PM10 exhibited a linear relationship, thus corroborating the appropriateness of the linear Poisson regression approach in Eq. 2. Moreover, including a further two-dimensional tensor product spline with basis dimension *k* = 5^2^ = 25 representing the interaction between *UTCI* and air pollution did not improve the predictive model significantly, as indicated by the corresponding non-significant likelihood ratio test results for *UTCI* in combination with PM2.5 (*p* = 0.60) and PM10 (*p* = 0.11), respectively. Thus, the main effects linear Poisson regression model adequately fitted our data concerning the influence of air pollution and biometeorological conditions on excess ILI incidence.

Regarding the influence of PM2.5 or PM10 and *UTCI* on ILI incidence for different age groups, the corresponding results after applying the models in Eqs. 2 and 3 were similar to those from the total counts, with the exception for the youngest age group (0–4 years) (Tab. S1 and Tab. S2). For young children, the estimates were smaller, and both averaged and net effects for PM2.5 or PM10 and *UTCI* were non-significant. The strongest effects of air pollution and biothermal conditions on ILI rates with the narrowest confidence intervals were observed for people 15–64 years, which can be attributed to the increased statistical power for this group (Armstrong et al. 2020) with the highest raw counts in the greatest sub-population (Fig. 1a and d).

**Fig. 3:**
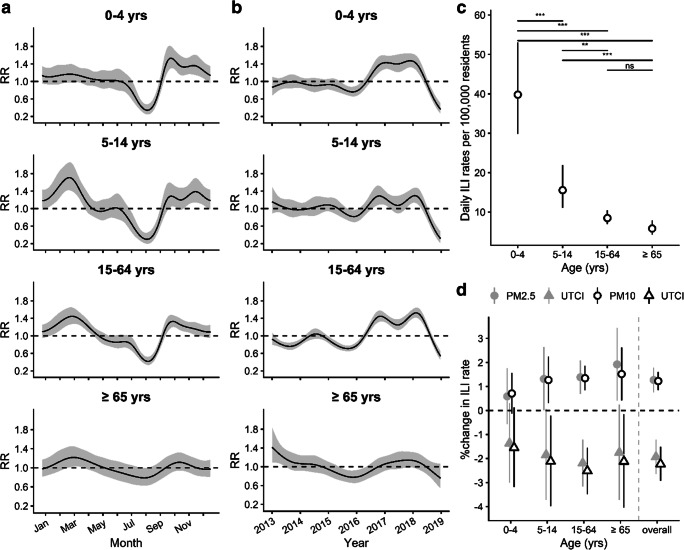
Seasonal (**a**) and long-term trends (**b**) from Poisson regression fitted by GAM for the combined age group series expressed as rate ratios (RR) relative to the overall mean. Estimated marginal mean ILI rates for the age groups (**c**) with tests for pairwise differences (ns: *p* ≥ 0.05, *: *p* < 0.05, **: *p* < 0.01, ***: *p* < 0.001) adjusted by Tukey method for comparing a family of 4 estimates. Interaction of the percentage changes in ILI rates per unit increase in the predictors *UTCI* and PM2.5 (grey symbols), or *UTCI* and PM10 (black open symbols), respectively, with age together with the overall estimate from the main effects model (**d**). Error bands and bars indicate 95% confidence intervals

Subsequently, the potential moderating influence of age on seasonal and annual trends, along with ILI incidence rate changes, was investigated. Figure 3 addresses the effects of the averaged lagged values of PM2.5 or PM10, respectively, combined with *UTCI*, and estimated from the pooled series of ILI counts for the four age groups. The seasonal (Fig. 3a) and annual trends (Fig. 3b) were alike those for the total series (Fig. 2), except for the oldest people (≥ 65 years), who showed less pronounced seasonal and long-term morbidity trends, compared to the other age groups. There was a statistically significant main effect with ILI rates decreasing with age, with post hoc tests indicating that all group differences, except that between the two oldest groups, were significant (Fig. 3c). The main overall effects of PM2.5 and *UTCI* or PM10 and *UTCI* (Fig. 3d) were significant and similar in size to the previous analyses (Fig. 2). Although, the estimated effects for young children (0–4 years) were smaller, compared to the other groups, and the corresponding confidence intervals were overlapping with zero, the interactions of both UTCI and PM2.5 or PM10, respectively, with age were statistically non-significant as indicated by the *p* values of the corresponding interaction terms from likelihood ratio tests (p_age*UTCI_ = 0.84, p_age*PM10_ = 0.65 for model with predictors UTCI and PM10; p_age*UTCI_ = 0.87, p_age*PM2.510_ = 0.55 with predictors UTCI and PM2.5) (Wood 2006).

## Discussion

Influenza outbreaks are well synchronized in time in temperate climates; however, the timing and individual characteristics of epidemics change regionally (Wenger and Naumova 2010; Schanzer et al. 2011). Our results confirmed that in Warsaw (Poland), the highest ILI rates were observed in winter, which is typical for temperate regions (Lofgren et al. 2007). The rate of ILI incidences reported approximately weekly, recorded between 2013 and 2018, shows noticeable variation in magnitude for different years. Inter-annual variations of influenza epidemics could be partially explained by the virus type and strain that actually predominated in the population (Reichert et al. 2004). According to Bouvier and Palese (2008), influenza type A viruses are transmitted more efficiently than type B, causing higher morbidity. Due to considerable antigenic diversity of human influenza viruses, annual ILI incidence rates vary from year-to-year, being strongly affected by two concurrent mechanisms: the emergence of new influenza strains and population immunization against new pathogens (Truscott et al. 2012). Very low ILI trend values at the end of 2018 were presumably attributable to the closure of the majority of medical centres for Christmas, which is typical for health service in Poland. Similar limitations in access to medical services at the end of the year were observed in Greece by Nastos and Matzarakis (2006).

Numerous studies have confirmed that meteorological conditions are to some extent associated with respiratory tract infections, especially with influenza and influenza-like illnesses incidence (Lowen et al. 2007; Tamerius et al. 2011; Van Noort et al. 2012; Wang et al. 2017; Peci et al. 2019; Price et al. 2019). In our study, air temperature and vapour pressure were found to be strongly negatively correlated with ILI incidence (− 0.73), while the association with relative humidity was moderately positive (0.44). Similar relationships were also found with ILI rates in the Republic of Korea (Park et al. 2020) and with lower respiratory tract infection hospitalization rate in China (Liu et al. 2016). Weaker, but significant correlations were reported between air temperature and hospitalization of children < 16 years due to influenza type A and B (− 0.53 and − 0.33 respectively) in Germany by du Prel et al. (2009). The impact of particular meteorological variables on morbidity can be different depending on influenza type predominating in the population (Price et al. 2019).

Climate and weather have a strong impact on ILI incidence, but examining the effects of individual meteorological factors on viral activity can lead to incomplete conclusions (Lawrence 2005), if potential interactions between these factors are not considered (Roussel et al. 2016). According to our results, daily ILI rates were strongly associated with biothermal conditions (*r* = − 0.72), determined by mean values of *UTCI* index in Warsaw. It is worth mentioning that combining the effect of various atmospheric variables into one measure has not been common approach in previous studies on influenza and ILI morbidity environmental conditionings, and indeed correlation was basically the same when using mean air temperature instead of *UTCI*. Nevertheless, *UTCI* considers the influence of temperature, wind, humidity, and heat radiation on human physiology, and thus, it helps overcoming methodological issues in regression analyses related to multi-collinearity between meteorological variables, especially if lagged values are considered. To our knowledge, so far, only Romaszko et al. (2019) have applied this biometeorological index in an epidemiological study and found strong inverse correlation (− 0.757) between weekly mean *UTCI* values and the number of hospital admissions for respiratory tract infections.

In our study, mean daily concentrations of PM2.5 and PM10 were moderately positively correlated with daily ILI rates (0.67 and 0.54, respectively). The observed associations were stronger than those reported previously by Su et al. (2019) in China (for PM2.5 and PM10 *r* = 0.31) or Park et al. (2020) in South Korea (for PM10 *r* = 0.38). Our results support the hypothesis that air pollution, especially particulate matter, could affect airways and increase susceptibility to respiratory virus infections (Ciencewicki and Jaspers 2007). Exposure to PM containing environmentally persistent free radicals (EPFRs) can induce pulmonary oxidative stress and suppress immune response to influenza virus (Lee et al. 2014). In addition, it has been recently suggested (Asadi et al. 2020) that influenza virus can spread through the airborne dust and other microscopic particles. Thus, high PM concentrations may increase the probability to inhale these pathogens and contribute to the infection development. Some researchers also interrelate high PM concentrations with the reduction of UV radiation reaching the ground, which inhibits biological activity of solar radiation and may contribute to the spread of influenza virus as a result (Qin et al. 2012).

We demonstrated that excess ILI incidence in Warsaw increased with rising PM2.5 or PM10 concentration, and with cooling biothermal conditions, indicated by decreasing *UTCI* values, although the observed effect proved to be smaller for the distributed model, with *UTCI* being insignificant as a predictor in the particular time lags. However, the net effect of *UTCI* and PM on ILI rates turned out to be statistically significant and nearly identical to the averaged effect of individual models. Our findings concerning *UTCI* as a predictor of ILI incidence are in accordance with conclusions by Romaszko et al. (2019), regarding respiratory tract infections. In that study, a significant increase in morbidity was observed, when *UTCI* values dropped by two thermal load classes, particularly when cold stress appeared at the same time. Our results acknowledged that excessive cooling of the human body requires a lot of energy for thermoregulation at the expense of immune defence, which makes an organism more susceptible to viral infections (Lofgren et al. 2007; Jaakkola et al. 2014). Concurrently, we found that overall RR of PM2.5 and PM10 affecting ILI rates in both the individual and the distributed models were very similar to the results of Su et al. (2019) obtained from quasi-Poisson regression models for single pollutants in China. On the contrary, Liu et al. (2019) observed that PM10 was negatively associated with weekly ILI medical consultations, while relationship between PM2.5 and ILI cases was positive. Moreover, Feng et al. (2016) noticed that the effect of PM2.5 on the ILI risk increased progressively with rising air pollution only in the flu season, when hosts were more susceptible to infection due to body cooling, and became insignificant in warmer and more humid conditions. Our data, which were appropriately described by a main effects linear Poisson regression model, suggest that high levels of air pollution and low *UTCI* values independently add to an increased ILI incidence rate. Although we did not find a higher sensitivity to PM in the cold season as reported by Feng et al. (2016), both studies support the idea that particular matter impact on influenza morbidity should be analysed together with biothermal conditions.

Another important finding of the present study was that PM concentration has the strongest effect on ILI incidence on the actual reporting period. Although data aggregation leads to smoothing extreme values and may result in not exact reflection of the actual exposure on the citizens, it has been previously demonstrated that weekly averaged data can be successfully used to assess influence of meteorological parameters and air pollution on ILI incidence (Huang et al. 2017; Liu et al. 2019).

In case of *UTCI*, there is less differentiation in the effect over the considered three periods, although the highest RR of increase in ILI was observed for 1-period lag. Regarding respiratory tract infections, previous studies confirmed that the number of daily consultations demonstrated a 7-day lag in the increased number of admissions after significant change in meteorological conditions (Romaszko et al. 2019); however, 10 and 14 days lags between worsened weather and higher morbidity were also reported (Nastos and Matzarakis 2006; Mäkinen et al. 2009).

For influenza type A incidence in Sweden, Sundell et al. (2016) found a significant association with the drop of air temperature the week before, what was in accordance with other studies on the effects of meteorological factors (i.e. air temperature and humidity) on influenza seasonality, recognizing 1-week lag between the cause and the effect (Wang et al. 2017; Park et al. 2020). However, longer incubation periods of influenza virus were also observed, e.g. 8 days in Japan (Tsuchihashi et al. 2011) and 13 days in China (Zhang et al. 2015). Likewise, recent studies confirm our results concerning short lagged effect of PM on ILI incidence. The impact of high concentration of PM2.5 and PM10 on higher ILI morbidity was limited usually up to 3 days before medical consultation (Huang et al. 2016; Su et al. 2019). Similar time lags between air pollution and increased number of medical consultations were reported for respiratory tract infections (Ostro et al. 1999; Tam et al. 2014).

In our study, the majority of ILI cases were reported among people 15–64 years, but when standardized for population size, daily ILI incidence rate showed a decreasing trend with age. The observed tendency could support the hypothesis that particularly children of 0–4 years are susceptible to virus infections, as their immunological system is not fully mature (Valenciano et al. 2013; Feng et al. 2016). Moreover, children are considered more vulnerable to the effects of meteorological factors and air pollution, as they tend to spend more time playing outdoors (Xu et al. 2013; Li et al. 2018). Contrarily, several studies (Feng et al. 2016; Huang et al. 2016; Su et al. 2019) found that ILI rates could be the lowest in the youngest age group due to the fact that infants were better protected and less frequently exposed to adverse atmospheric conditions. Furthermore, ILI rates for young children in our data could possibly be overestimated, as young organisms sometimes manifest non-specific febrile illnesses or suffer from other respiratory diseases that are very difficult to distinguish from ILI (Cox and Subbarao 1999). We also observed that ILI rates in the oldest age group (≥ 65 years) were the smallest and less diversified seasonally and annually. This finding is in accordance with a previous study from Australia, where the smallest effect of seasonality on influenza risk among elderly people was associated with a high vaccine uptake by this age group (Huang et al. 2017). In Poland, people aged over 65 years were the most immunized population group (Brydak et al. 2012), although the rate of influenza vaccination in the whole Polish society was in general very low comparing to other European countries (ECDC 2018). Concurrently elderly people were reported to have less social interactions than school children and working adults, which resulted in lower contact rates and could limit the spread of infection within the oldest citizens (Wallinga et al. 2006; Mossong et al. 2008). On the other hand, increased social contacts might not be the likely explanation for the higher ILI rates in young children (0–4 years), as typically in Poland, until 3 years old, they usually spend time at home with mothers or babysitters. In 2017, only 11.6% of children under age 3 in Poland attended early childhood education and care institutions (Eurydice 2019).

In our study, ILI rates significantly declined with age, when adjusted for population size, nevertheless the effects of PM2.5 or PM10 and *UTCI* on daily ILI rates in Warsaw did not depend on age. Therefore, our results confirm observation of Roussel et al. (2016) that climatic factors have a uniform impact on influenza spread within the population regardless of age.

Due to the nature of ILI data collected by Polish Sanitary Inspection, our study has some limitations. First, ILI reports are anonymised, and it is impossible to determine whether individuals included in the statistics lived in the study area and were influenced by the investigated atmospheric factors or arrived from different places (Price et al. 2019). Second, as discussed above, our data was aggregated to reporting periods; thus, meteorological parameters had to be averaged accordingly, what smoothed the extremums of atmospheric conditions and reduced their possible impact on influenza morbidity. In addition, in winter, when ILI morbidity is the greatest, people spend a lot of time indoors, where biothermal conditions and air quality differ significantly from those outdoors. Therefore, standard meteorological and air pollution data may be an imperfect indicator of individual exposure to particular environmental conditions (Chan et al. 2009; Wiemken et al. 2017). Finally, as proven recently, even laboratory-infected volunteers do not regularly demonstrate all symptoms of ILI (Babcock et al. 2006; Lau et al. 2010), which may suggest that prevalence of influenza infections in the studied population could be even higher than reported ILI rates (Van Noort et al. 2012).

Nevertheless, although data aggregation leads to smoothing extreme values and may result in not exact reflection of the actual exposure on the citizens, it has been previously demonstrated that weekly averaged data can be successfully used to assess the influence of meteorological parameters and air pollution on ILI incidence (Huang et al. 2017; Liu et al. 2019).

## Conclusion

Our results confirmed that there is a significant relationship between ILI incidence in Warsaw and biothermal conditions, as well as PM concentration after adjusting for seasonal and annual trends. We also showed that *UTCI* can serve as predictor not only of respiratory tract infections (Romaszko et al. 2019) in general but also of influenza-like illnesses. Nevertheless, taking into account seasonal and annual trends in such analysis is advisable, as virus activity in the population is also affected by other factors, e.g. seasonally changing host susceptibility, contact rates among people, and pathogen infectivity connected with antigenic novelty of virus strains (Lofgren et al. 2007; Van Noort et al. 2012; Li et al. 2018).

This study concerning the impact of biometeorological factors and air pollution on the morbidity due to influenza-like illnesses in Warsaw extends former research focused only on the occurrence of influenza in the whole Mazovia Voivodeship during two epidemiological seasons (Korzeniecki 2015). Better understanding environmental conditionings of influenza seasonality in temperate climate will be beneficial to forecasting future dynamics of ILI and to planning clinical and public health resources under climate change or pandemic scenarios (Watts et al. 2015).

## Supplementary information

ESM 1 (PDF 947 kb)
