# Supplementary material for: Impact of biometeorological conditions and air pollution on influenza-like illnesses incidence in Warsaw
Source: Int J Biometeorol. 2021 Jan 17;65(6):929–44. doi: 10.1007/s00484-021-02076-2 (PMC8149351; doi:10.1007/s00484-021-02076-2)
Supplement: Supplementary file 1 — (PDF 947 kb) [file 484_2021_2076_MOESM1_ESM.pdf]

Electronic Supplemental Material (ESM) accompanying the manuscript

Impact of biometeorological conditions and air pollution on influenza-like illnesses incidence in Warsaw

Katarzyna Lindner-Cendrowska[1] and Peter Bröde[2]

[1] Institute of Geography and Spatial Organization, Polish Academy of Sciences, Twarda 51/55, 00-818, Warsaw, Poland, klindner@twarda.pan.pl, ORCID 0000-0002-8322-4653

[2] Leibniz Research Centre for Working Environment and Human Factors at TU Dortmund (IfADo), Dortmund, Germany, broede@ifado.de, ORCID 0000-0001-8107-704X

Revised submission to International Journal of Biometeorology 2020-11-05

Supplemental Note 1:

Calculation of mean radiant temperature (*tmrt*)

in BioKlima v2.6 software authored by **Prof. Krzysztof Błażejczyk, PhD** (k.blaz@twarda.pan.pl), available at https://www.igipz.pan.pl/bioklima.html (accessed 2020-07-21):

$$tmrt = \left(\frac{\frac{R}{Irc} + 0.5 \cdot Lg + 0.5 \cdot La}{s_h \cdot \sigma} \right)^{0.25} - 273$$

where:

R – absorbed solar radiation (W/m^2) was calculated using the *SolAlt* model on the basis of information concerning total cloud cover, elevation of the sun and albedo of clothing (see below).

Irc **–** the coefficient reducing convective and radiative heat transfer through clothing (see formula below)

Lg – ground radiation (W/m^2)

La — atmosphere outgoing radiation (W/m^2)

s_h — emissivity coefficient for humans (0.95)

σ — the Stefan-Boltzmann constant (5.667/10^8 W/m^2·K^4)

SolAlt model to calculate **R**:

- for $hSl \leq 4°$ $$\boldsymbol{R = (1.642 + 0.254 \cdot hSl)^2 \cdot (1 - 0.01 \cdot ac) \cdot Irc}$$
- for $hSl > 4°$ and $N \leq 20\%$ $$\boldsymbol{R = (103.573 \cdot \ln(hSl) - 140.6) \cdot (1 - 0.01 \cdot ac) \cdot Irc}$$
- for $hSl > 4°$ and N = 21-50% $$\boldsymbol{R = 1.4 \cdot e^{\left(5.383 - \frac{16.072}{hSl}\right)} \cdot (1 - 0.01 \cdot ac) \cdot Irc}$$
- for $hSl > 4°$ and N = 51-80% $$\boldsymbol{R = 1.4 \cdot e^{\left(5.012 - \frac{11.805}{hSl}\right)} \cdot (1 - 0.01 \cdot ac) \cdot Irc}$$
- for $hSl > 4°$ and $N > 80\%$ $$\boldsymbol{R = 0.9506 \cdot hSl^{1.039} \cdot (1 - 0.01 \cdot ac) \cdot Irc}$$
 (also for shaded sites when $hSl > 4°$)

hSl – sun altitude (°)
N – cloudiness (%)
ac – mean albedo of clothing and skin (30%)

$$\boldsymbol{Irc = \frac{hc'}{hc' + hc + 21.55 \cdot 10^{-8} \cdot T^3}}$$

T – air temperature (K)
hc – coefficient of convective and radiative heat transfer (K/W·m^2)

$$hc = (0.013 \cdot p - 0.04 \cdot t - 0.503) \cdot (v + v')^{0.4}$$

hc' – coefficient of heat transfer through clothing (K/W·m^2)

$$hc' = \frac{(0.013 \cdot p - 0.04 \cdot t - 0.503) \cdot 0.53}{Icl \cdot [1 - 0.27 \cdot (v + v')^{0.4}]}$$

p – air pressure (hPa)
t – air temperature (°C)
v – wind speed (m/s)
v' – velocity of man motion (1.1 m/s)
Icl – clothing insulation (clo) $Icl = 1.691 - 0.0436 \cdot t$
[note: at $t < -30$°C Icl = 3.0 clo, and at $t > 25$°C Icl = 0.6 clo]

$$\boldsymbol{Lg = s \cdot \sigma \cdot (273 + tg)^4}$$

s – emissivity coefficient (0.97 for natural surfaces)
σ – the Stefan-Boltzmann constant (5.667/10^8 W/m^2·K^4)
tg – ground surface temperature (°C)
- for $N \geq 80\%$ $tg = t$
- for $N < 80\%$ and $t \geq 0$°C $tg = 1.25 \cdot t$
- for $N < 80$ and $t < 0$°C $tg = 0.9 \cdot t$

$$\boldsymbol{La = s \cdot \sigma \cdot (273 + t)^4 \cdot (0.82 - 0.25 \cdot 10^{-0.094 \cdot vp})}$$

vp – vapour pressure (hPa)

Supplemental Tables

Table S1: Percentage change in ILI incidence rates per unit increase in the predictors, with 95% confidence intervals (CI) in brackets, estimated by **individual lag models** adjusting for seasonal and annual trends. Different models were fitted using zero-, one- or two-period lags as well as the averaged lagged values (avg) of *UTCI* together with PM2.5 (left panel) or PM10 (right panel), respectively, as predictors. Results are presented for the different age groups and the total sample.

		% change (95%-CI) in ILI rates per unit increase			
groups	***lag***	***UTCI (°C)***	***PM2.5 (μg/m³)***	***UTCI (°C)***	***PM10 (μg/m³)***
total	0	-0.62 (-1.52, 0.28)	0.85 (0.27, 1.44)**	-0.79 (-1.69, 0.11)	0.78 (0.35, 1.21)***
	1	-1.27 (-2.16,-0.38)**	0.33 (-0.26, 0.91)	-1.37 (-2.25,-0.48)**	0.41 (-0.02, 0.85)
	2	-0.78 (-1.69, 0.15)	0.32 (-0.26, 0.89)	-0.84 (-1.76, 0.10)	0.27 (-0.16, 0.71)
	avg	-1.88 (-3.20,-0.54)**	1.22 (0.27, 2.18)*	-2.17 (-3.49,-0.83)**	1.20 (0.51, 1.89)***
0-4 yrs	0	-0.25 (-1.28, 0.79)	0.55 (-0.11, 1.22)	-0.37 (-1.40, 0.68)	0.51 (0.02, 1.00)*
	1	-1.12 (-2.12,-0.11)*	0.18 (-0.48, 0.85)	-1.20 (-2.21,-0.19)*	0.32 (-0.17, 0.82)
	2	-0.49 (-1.53, 0.56)	0.02 (-0.62, 0.68)	-0.50 (-1.55, 0.56)	0.04 (-0.44, 0.54)
	avg	-1.35 (-2.88, 0.20)	0.59 (-0.48, 1.67)	-1.54 (-3.07, 0.02)	0.71 (-0.08, 1.50)
5-14 yrs	0	-0.76 (-1.81, 0.29)	0.59 (-0.09, 1.27)	-0.87 (-1.91, 0.18)	0.56 (0.06, 1.08)*
	1	-1.33 (-2.35,-0.30)*	0.53 (-0.13, 1.20)	-1.44 (-2.46,-0.42)**	0.62 (0.12, 1.12)*
	2	-0.48 (-1.55, 0.61)	0.47 (-0.17, 1.12)	-0.55 (-1.62, 0.53)	0.39 (-0.11, 0.88)
	avg	-1.81 (-3.35,-0.24)*	1.31 (0.24, 2.40)*	-2.08 (-3.61,-0.52)**	1.27 (0.48, 2.07)**
15-64 yrs	0	-0.78 (-1.73, 0.18)	1.04 (0.42, 1.66)***	-0.97 (-1.92,-0.02)*	0.93 (0.48, 1.39)***
	1	-1.44 (-2.37,-0.50)**	0.34 (-0.28, 0.96)	-1.53 (-2.46,-0.59)**	0.41 (-0.06, 0.87)
	2	-0.99 (-1.96,-0.02)*	0.33 (-0.28, 0.94)	-1.05 (-2.03,-0.07)*	0.29 (-0.17, 0.75)
	avg	-2.25 (-3.64,-0.84)**	1.38 (0.38, 2.40)**	-2.56 (-3.94,-1.16)***	1.33 (0.60, 2.06)***
≥ 65 yrs	0	-0.42 (-1.36, 0.52)	0.81 (0.20, 1.43)**	-0.63 (-1.56, 0.31)	0.78 (0.33, 1.24)***
	1	-0.70 (-1.63, 0.24)	0.27 (-0.34, 0.89)	-0.79 (-1.72, 0.16)	0.33 (-0.14, 0.79)
	2	-0.68 (-1.62, 0.27)	0.60 (0.01, 1.20)*	-0.78 (-1.73, 0.18)	0.44 (-0.01, 0.90)
	avg	-1.19 (-2.56, 0.19)	1.48 (0.48, 2.50)**	-1.55 (-2.92,-0.17)*	1.30 (0.58, 2.03)***

Notes: *avg*: averaged lagged values; *** $p < 0.001$, ** $p < 0.01$, * $p < 0.05$

Table S2: Percentage change in ILI incidence rates per unit increase in the predictors, with 95% confidence intervals (CI) in brackets, estimated by the **distributed lag model** adjusting for seasonal and annual trends. Models simultaneously used zero-, one- and two-period lagged values of *UTCI* together with PM2.5 (left panel) or PM10 (right panel), respectively, as predictors. Results also present the overall net effect, calculated from the summed coefficients of the lagged values, for the different age groups and the total sample.

		% change (95%-CI) in ILI rates per unit increase			
groups	***lag***	***UTCI (°C)***	***PM2.5 (μg/m³)***	***UTCI (°C)***	***PM10 (μg/m³)***
total	0	-0.45 (-1.40, 0.51)	0.67 (0.07, 1.28)*	-0.63 (-1.59, 0.33)	0.65 (0.21, 1.10)**
	1	-0.84 (-1.84, 0.18)	0.23 (-0.38, 0.84)	-0.88 (-1.88, 0.13)	0.33 (-0.12, 0.79)
	2	-0.46 (-1.43, 0.53)	0.31 (-0.26, 0.89)	-0.49 (-1.46, 0.48)	0.22 (-0.22, 0.65)
	net	-1.73 (-3.10,-0.35)*	1.22 (0.26, 2.20)*	-1.99 (-3.34,-0.63)**	1.20 (0.51, 1.91)***
0-4 yrs	0	-0.02 (-1.11, 1.09)	0.39 (-0.29, 1.08)	-0.15 (-1.26, 0.96)	0.40 (-0.10, 0.91)
	1	-0.93 (-2.07, 0.22)	0.13 (-0.56, 0.83)	-0.98 (-2.12, 0.17)	0.28 (-0.23, 0.80)
	2	-0.17 (-1.29, 0.96)	0.04 (-0.62, 0.71)	-0.13 (-1.25, 1.00)	0.02 (-0.48, 0.52)
	net	-1.12 (-2.69, 0.48)	0.57 (-0.52, 1.67)	-1.27 (-2.84, 0.33)	0.71 (-0.09, 1.51)
5-14 yrs	0	-0.54 (-1.64, 0.57)	0.35 (-0.35, 1.05)	-0.67 (-1.77, 0.45)	0.37 (-0.16, 0.89)
	1	-1.14 (-2.30, 0.04)	0.51 (-0.18, 1.21)	-1.21 (-2.36,-0.04)*	0.60 (0.07, 1.12)*
	2	-0.01 (-1.15, 1.15)	0.49 (-0.16, 1.16)	-0.04 (-1.18, 1.10)	0.35 (-0.16, 0.86)
	net	-1.68 (-3.27,-0.05)*	1.36 (0.26, 2.47)*	-1.91 (-3.49,-0.31)*	1.32 (0.51, 2.13)**
15-64 yrs	0	-0.61 (-1.61, 0.40)	0.86 (0.22, 1.50)**	-0.82 (-1.81, 0.19)	0.80 (0.34, 1.27)***
	1	-0.82 (-1.88, 0.24)	0.21 (-0.44, 0.85)	-0.86 (-1.91, 0.19)	0.30 (-0.18, 0.78)
	2	-0.68 (-1.71, 0.36)	0.31 (-0.30, 0.92)	-0.73 (-1.74, 0.30)	0.22 (-0.24, 0.68)
	net	-2.10 (-3.52,-0.65)**	1.38 (0.36, 2.41)**	-2.39 (-3.80,-0.96)**	1.33 (0.59, 2.06)***
≥ 65 yrs	0	-0.43 (-1.43, 0.57)	0.71 (0.08, 1.35)*	-0.63 (-1.62, 0.37)	0.70 (0.24, 1.17)**
	1	-0.29 (-1.35, 0.79)	0.15 (-0.48, 0.80)	-0.29 (-1.35, 0.78)	0.21 (-0.27, 0.69)
	2	-0.52 (-1.54, 0.51)	0.59 (-0.01, 1.20)	-0.63 (-1.63, 0.39)	0.38 (-0.08, 0.83)
	net	-1.24 (-2.64, 0.19)	1.46 (0.44, 2.50)**	-1.54 (-2.94,-0.13)*	1.29 (0.56, 2.03)***

Notes: *net*: net effect from summed coefficients for lagged values; *** $p < 0.001$, ** $p < 0.01$, * $p < 0.05$

Supplemental Figures

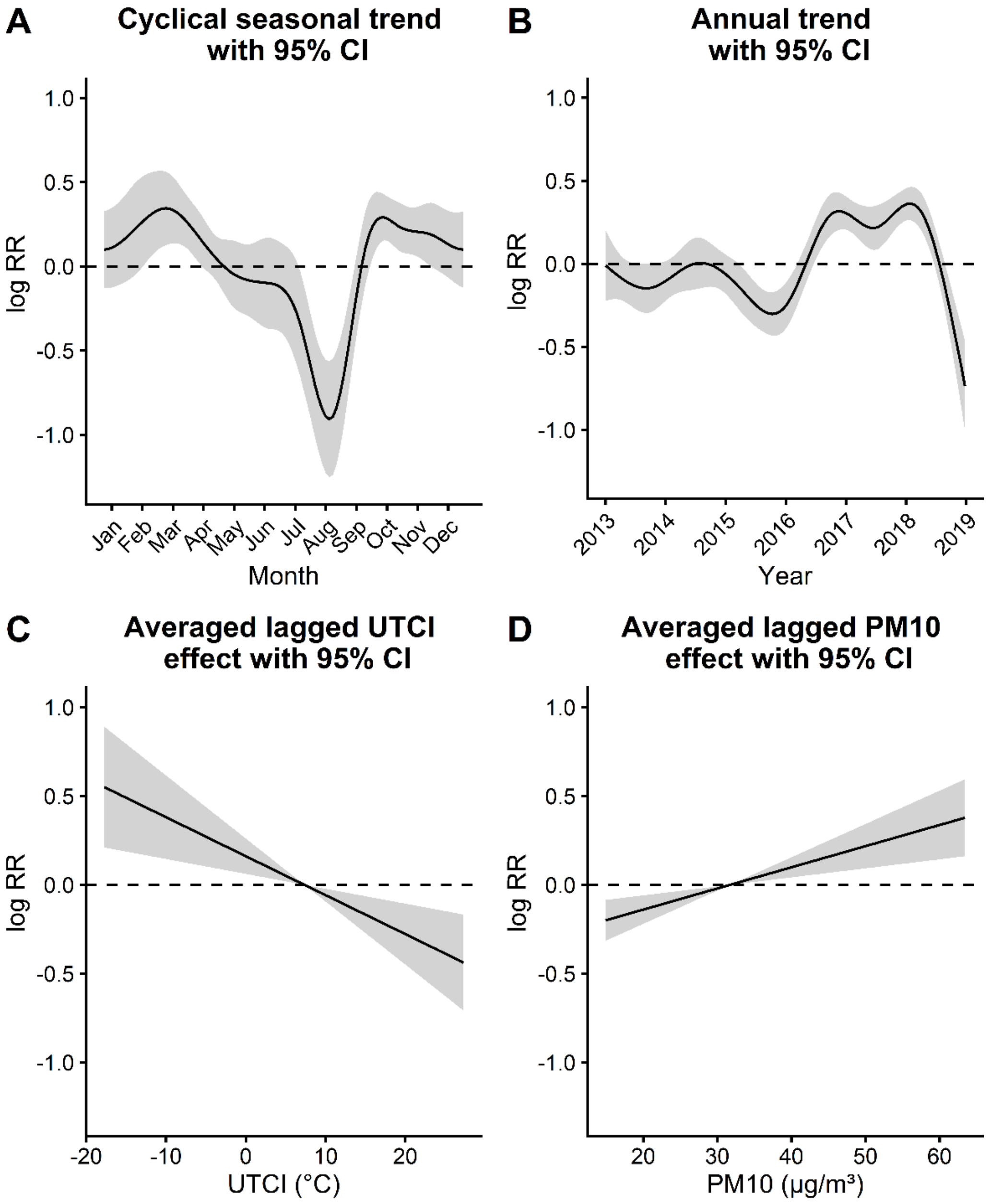

Figure S1: Generalized additive model (GAM) predictions for total daily ILI rates expressed as log rate ratios (RR) relative to the overall mean. Fitted cubic regression spline functions representing seasonal (**A**) and long-term annual trends (**B**), as well as tensor product spline functions allowing for potential nonlinear effects of the averaged lagged values of *UTCI* (**C**) and PM10 (**D**), respectively. Error bands indicate 95% confidence intervals (CI).
